# Prevalence of suicidal behaviour among nursing and midwifery college students in Ghana

**DOI:** 10.1002/nop2.271

**Published:** 2019-04-09

**Authors:** Emmanuel Nii‐Boye Quarshie, Haziel Vera Cheataa‐Plange, Francis Annor, Winifred Asare‐Doku, Joshua King Safo Lartey

**Affiliations:** ^1^ Department of Psychology University of Ghana Legon Ghana; ^2^ Centre for Suicide and Violence Research (CSVR) Accra Ghana; ^3^ School of Psychology University of Leeds Leeds UK; ^4^ Nursing and Midwifery Training College Korle‐Bu Accra Ghana; ^5^ The University of Newcastle Callaghan New South Wales Australia; ^6^ Business School University of Ghana Legon Ghana

**Keywords:** Ghana, midwifery, nurses, students, suicidal behaviour

## Abstract

**Aim:**

To provide exploratory and descriptive evidence on the prevalence estimate and some demographic correlates of suicidal behaviour among nursing and midwifery college students in Ghana.

**Design:**

We used a cross‐sectional survey design.

**Method:**

An anonymous survey involving a randomly selected sample of 305 nursing and midwifery college students was conducted in March–May 2017. The Suicide Behavior Questionnaire‐Revised was used to assess suicidal behaviour (i.e., ideation, planning, threat and attempt) and suicidal behaviour risk.

**Results:**

The lifetime prevalence of suicide ideations (15.4%; 95% confidence interval [CI] = 0.11–0.20), plans (6.6%; 95% CI = 0.04–0.10), attempts (2.3%; 95% CI = 0.01–0.05), threats (13.4%; 95% CI = 0.10–0.18) and 12‐month prevalence of ideations (21.3%; 95% CI = 0.17–0.26) are comparable to estimates found in both high‐income and low‐ and middle‐income countries. However, associations between the demographic variables studied and suicidal behaviour risk were not statistically significant.

## 1 INTRODUCTION

Suicidal behaviour—defined in this study as including suicidal thoughts (ideation), plans (planning), threats and attempts to kill oneself—has gained increased public concern, as the phenomenon represents a serious global public health challenge (WHO, 2014). Suicide is the second leading cause of death among persons aged between 15–29 years, and it has been estimated to claim 1.5 million lives globally between 2004 and the year 2020 (WHO, 2004,2014). Presently, in Ghana, local media reports are showing frequent cases of suicide. Predominantly, these suicide reports in the media involve youth and students at various levels of education—junior high school, senior high school, college and university (Daily Graphic, 2017; Dailyguide Africa, 2017a,2017b; Frimpong, 2017). Again, recent anecdotal reports (Dailyguide Africa, 2016; Kubi, 2017; Nyav, 2015) and findings from a systematic content analysis of recent media reports (see Quarshie, Osafo, Akotia, & Peprah, 2015) show an increasing trend of suicide in youth and students in Ghana.

Suicide is stigmatized in most countries around the world (WHO, 2014). However, Ghana is a religious country (71.2% Christian and 17.6% Islam; Ghana Statistical Service, 2013) where suicide is fiercely tabooed and (attempted suicide is) criminalized, with no official database or national formal statistics on the prevalence, trends and characteristics of the phenomenon in the country (Osafo, Akotia, Andoh‐Arthur, & Quarshie, 2015). Although some previous studies from Ghana (Eshun, 2003; Knizek, Akotia & Hjelmeland, 2011; Osafo, Hjelmeland, Akotia, & Knizek, 2011) have explored the socio‐cultural determinants of suicidal ideation and the attitudes of university students towards suicide, generally, primary research examining the prevalence, correlates, risks and protective factors related to suicidal behaviour among adolescents and youth in Ghana remains scant (Asante, Kugbey, Osafo, Quarshie, & Sarfo, 2017; Quarshie et al., 2015). More pointedly, primary studies with focus on the phenomenon in college nursing and midwifery students in Ghana are unavailable, although there are local media reports of suicidality among this category of students (Frimpong, 2017).

Globally, recent studies have widely reported mental disorders and suicidal behaviours among university and college students in general, with suicidal ideations and other related mental health challenges being more frequent in females and adolescents (Amanya, Nakitende, & Ngabirano, 2018; Auerbach et al., 2016; Eskin et al., 2016; Han et al., 2016; Li et al., 2014; Mortier et al., 2015,2017; Okwaraji & En, 2014; Yang, Zhang, Sun, Sun, & Ye, 2015). However, studies with focus on estimating the prevalence and determining the socio‐demographic factors that present as correlates, risks or protective factors related to suicidal behaviours specifically among nursing (and midwifery) students are generally inadequate. Across the continent of Africa, there is a lack of published studies on the prevalence of suicidal behaviours among college students (Cipriano, Cella, & Cotrufo, 2017; Eshun, 2003; Franklin et al., 2017; Mortier, Cuijpers et al., 2018). Therefore, several previous studies conducted mainly in high‐income countries and a few studies among students from Africa have been reviewed to contextualize the present study.

Eshun (2003) compared samples of college students in Ghana and America on the socio‐cultural determinants of self‐reported suicidal ideation. The findings showed among other things that no statistically significant relationship exists between religiosity and suicidal ideation in both samples, even though female gender showed a statistically significant association with suicidal ideation in the Ghanaian sample (Eshun, 2003). In South Africa, Van Niekerk, Scribante, and Raubenheimer (2012) surveyed 874 medical students from three universities. In terms of lifetime prevalence estimates, 32.3% of the students reported suicidal ideation, while 6.9% reported suicidal attempt. Van Niekerk et al. (2012) found no statistically significant evidence in terms of the association between suicidal attempt and age, gender, relationship status, place of study or year of study. In a cross‐sectional survey of 1,130 Portuguese nursing students, Leal and Santos (2016) found a lifetime prevalence of suicidal behaviour of 5.22% (female = 5.43%; male = 4.21%), with no significant statistical difference across the years of study. Similarly, Aradilla‐Herrero, Tomás‐Sábado, and Gómez‐Benito (2014) observed an overall suicide risk of 14% among nursing students in Spain. Of the 93 students surveyed, 14% had thought about suicide at some point in their lives and 6.5% had previously made a suicide attempt, with a statistically significant negative association observed between self‐esteem and suicide risk (Aradilla‐Herrero et al., 2014). Among nursing students in Greece, Melissa‐Halikiopoulou, Tsiga, Khachatryan, and Papazisis (2011) reported a lifetime suicide ideation prevalence estimate of 10.6% (females = 8%; males = 25%), with 1.7% female participants expressing the desire to actually die by suicide if they had the chance. Also, a statistically significant positive association between suicidal ideations and depression was found (Melissa‐Halikiopoulou et al., 2011). In a longitudinal study of the predictors of suicidal ideation among college students, Nam, Hilimire, Jahn, Lehmann, and DeVylder (2018) reported that age, gender and sexual orientation showed no statistically significant association with suicidal ideation, whereas race/ethnicity showed significant association with suicidal ideation intensity. Also, truancy has been found as a strong correlate of suicidal behaviour among students (Asante et al., 2017).

More recently, in the WHO World Mental Health International College Student Project, cross‐sectional web‐based self‐report questionnaires were administered to 13,984 first‐year students across 19 colleges in eight countries: Australia, Belgium, Germany, Mexico, Northern Ireland, South Africa, Spain and the United States (Mortier, Auerbach, Alonso, Bantjes et al., 2018). In terms of lifetime prevalence, 32.7% of the students reported suicidal ideation, 17.5% reported suicidal plans and 4.3% reported suicide attempts. About 12‐month prevalence, 17.2% of the students reported suicidal ideation, 8.8% reported suicidal plans and 1.0% reported suicide attempts. The study further showed non‐heterosexual orientation and heterosexual orientation with some same‐sex attraction as the strongest correlates of suicidal thoughts and behaviours across the overall sample (Mortier, Auerbach, Alonso, Bantjes et al., 2018).

Globally, the most recent meta‐analysis of prevalence studies reporting suicidal behaviours among college students has found various pooled prevalence estimates of the behaviour in college students (Mortier, Cuijpers et al., 2018): lifetime suicidal ideation (22.3% [95% confidence interval (CI) 19.5%–25.3%]), plans (6.1% [95% CI 4.8%–7.7%]) and attempts (3.2% [95% CI 2.2%–4.5%]). The 12‐month prevalence estimates were 10.6% (95% CI 9.1%–12.3%), 3.0% (95% CI 2.1%–4.0%) and 1.2% (95% CI 0.8%–1.6%), for suicidal ideation, plans and attempts, respectively (Mortier, Cuijpers et al., 2018).

Thus far in Ghana, only three published primary studies are available on the prevalence and correlates of suicidal behaviour among senior high school (SHS) students (Asante et al., 2017; Baiden et al., 2019; Liu, Huang, & Liu, 2018), with no published study on the phenomenon among (nursing and midwifery) college students, although media reports are showing worrying trends of suicidal deaths among students across the country (Quarshie et al., 2015). A college in Ghana is an immediate post‐senior high school level/stage of education. Hence, a potential contribution of the present study is the provision of a comparative evidence to help assess the trends of the prevalence of suicidal behaviour as young people move from SHS to college.

The present study is thus born out of the need for systematic evidence on the prevalence and (demographic) correlates of suicidal behaviour among college trainee nurses and midwives in Ghana for a pioneering understanding of the extent of the problem among this population and to potentially inform intervention and prevention efforts and programmes in the country. Specifically, this study seeks to provide exploratory, descriptive evidence on the prevalence estimates of suicidal behaviour (ideations, planning, threats and attempts) and the associations between some socio‐demographic factors and suicidal behaviour risk among nursing and midwifery college students in Ghana.

### 1.1 Structure of nursing education in Ghana

Ghana is a West African country with a nurse‐population ratio of 1:542 (Ghana Health Service, 2017). Being the first to achieve independence from colonial rule, Ghana led the development of nurses’ education, with the establishment of the first university‐based diploma programme to train nurses in tropical Africa (Opare & Mill, 2000). Detailed historical facts and contemporary development of nursing and midwifery training in Ghana have been provided elsewhere (see Adu‐Gyamfi & Brenya, 2016; Bell, Rominski, Bam, Donkor, & Lori, 2013; Bvumbwe & Mtshali, 2018; Opare & Mill, 2000; Talley, 2006). The nursing and midwifery professions remain female‐dominated in Ghana and as such fewer males enrol as trainees (Adu‐Gyamfi & Brenya, 2016; Tagoe & Quarshie, 2017; Talley, 2006). Although some universities in Ghana offer nursing and midwifery training programmes, most nurses and midwives in the country are educated in training colleges often with a teaching hospital affiliation (Bell et al., 2013). Currently, four basic nursing and midwifery programmes (Registered General Nursing, Registered midwifery, Registered Community Health Nursing and Registered Mental Health Nursing) are offered by universities and training colleges across the country (Nursing & Midwifery Council of Ghana – NMCG, 2018). Annually, about 400 persons graduate from nursing and midwifery training institutions in Ghana (Darko, 2015).

### 1.2 Theoretical framework

The theory guiding this study is the risk‐protective factor model (Monahan, Oesterle, Rhew, & Hawkins, 2014). Risk and protective factors play a critical role in suicide prevention and identifying risk, and protective factors provide critical information to assess and manage suicide risk in individuals. Socio‐demographic factors such as non‐heterosexual orientation, female gender, low socio‐economic status, first year of college, marital status, family relationships, adolescence, among others, have been found to be associated with increased odds and/or risk factors of suicidal behaviour (Aranmolate, Bogan, Hoard, & Mawson, 2017; Engin, Gurkan, Dulgerler, & Arabaci, 2009; Eshun, 2003; Fox et al., 2018; Hawton, Saunders, & O'Connor, 2012; Mortier, Auerbach, Alonso, Axinn et al., 2018; Mortier, Auerbach, Alonso, Bantjes et al., 2018; Mortier, Cuijpers et al., 2018; Mortier et al., 2017,2015; Tyssen, Vaglum, Grønvold, & Ekeberg, 2001). Some studies have shown religious involvement, being married and having supportive social connections (Colucci & Martin, 2008; Gearing & Alonzo, 2018; Milner et al., 2015) to be associated with reduced odds of suicidal behaviour and/or as protective factors against suicide ideation and attempts. However, in the present study, besides estimating the prevalence of suicidal behaviour, it is aimed to explore the associations between some socio‐demographic factors (i.e., age, gender, religious groups, ethnic groups, romantic relationship status, sexual orientation, marital status, year of study, programme of study, school residential status and truancy) and suicidal behaviour risk among nursing and midwifery college students in Ghana.

## 2 DESIGN AND METHODS

The general methodological approach to this study was descriptive in nature. Specifically, we used a cross‐sectional (prevalence) survey design (Lachat et al., 2016) involving the use of a standardized questionnaire. The survey was conducted between March and May 2017 at the Nurses and Midwifery Training College, Korle‐Bu, Accra, Ghana. Three hundred twenty (320) questionnaires were distributed out of which 305 questionnaires were returned with complete information, yielding a 95% response rate.

Table 1 shows the key demographic characteristics of the participants in this study. As of the time of this study, the student population of the institution of interest was 1,042. Thus, guided by the sample size determination table of Krejcie and Morgan (1970) a sample of 278 was computed (taken level of precision = 0.5, chi‐square for 1 degree of freedom = 3.841 and population proportion = 0.50). Additional 42 questionnaires were administered to make up for potential losses due to incompleteness and non‐return of questionnaires. For each level (year of study) programme, there were three classes. A class was randomly selected at each course level of study, and all the students in each randomly selected class qualified to respond to the survey. The 305 were made up of 277 females (90.8%) and 28 males (9.2%), reflecting the consistent disproportionate gender distribution of participants in previous studies among this group of students in Ghana (Tagoe & Quarshie, 2017). The participants were aged between 18–35 years, with a mean age of 22.49 years (*SD* = 2.39). Table 1 shows the socio‐demographic characteristics of the participants. Most participants self‐identified as Christian (94.4%), heterosexual (91.6%), single (94.4%) and enrolled on the Registered General Nurse (RGN) programme (52.5%).

**Table 1:** Socio‐demographic characteristics of participants (*N* = 305)

Characteristics	Frequency	%
Gender		
Female	277	90.8
Male	28	9.2
Religious groups		
Christian	287	94.4
Muslim	17	5.6
Ethnic groups		
Akan	138	46.2
Ewe	71	23.7
Ga‐Dangme	66	22.1
Guan	5	1.7
Other^a^	19	6.2
In romantic relationship		
No	157	52.2
Yes	144	47.8
Sexual orientation		
Heterosexual	241	91.6
Non‐heterosexual^b^	22	8.4
Marital status		
Single	288	94.4
Married	11	3.6
Other	6	2.0
Year of study		
1st year	82	26.9
2nd year	121	39.7
3rd year	102	33.4
Programme of study		
RGN	160	52.5
Midwifery	83	27.2
RCHN	62	20.3
School residential status		
Resident	257	84.3
Non‐resident	48	15.7
Truancy^c^		
≤5 days	288	94.7
>5 days	48	5.3

Notes

RCHN: Registered Community Health Nursing; RGN: Registered General Nursing.

a Ten response options were provided under ethnic groups; however, each of six options had frequencies <5, hence were grouped into an “other” category.

b A disproportionately higher number of the participants self‐identified as heterosexual, compared to lesbian, gay, bisexual and transgender orientations, which were collapsed into “non‐heterosexual.”

c Truancy refers to number of days absent from college without permission during the past 12 months.

### 2.1 Measures

The questionnaire used for this study was in English and consisted of two parts. The first part was made up of 11 items on socio‐demographic characteristics of the participants: age, gender, religious group, ethnic group, romantic relationship status, sexual orientation, marital status, years of study, programme of study, school residential status and truancy (see Table 1). The second part of the questionnaire was made up of the four‐item Suicide Behavior Questionnaire‐Revised (SBQ‐R) developed by Osman et al. (2001). The phrasing of each item was such that it asked about different aspects of suicidal behaviours (i.e., ideation/thoughts, plan/planning, threats and attempts). The first item explored whether the respondent had ever had thoughts of suicide or engaged in suicidal behaviour in his/her lifetime (i.e., *have you ever thought about or attempted to kill yourself?*). The second question evaluated how frequent, over the preceding 12 months, the respondent had been having ideations of suicide (i.e., *how often have you thought about killing yourself in the past year?*). The third question enquired about threats to engage in suicidal behaviour (i.e., *have you ever told someone that you were going to commit suicide, or that you might do it?*). The last question explored the participants’ self‐reported likelihood of engaging in a suicidal behaviour in the future (*how likely is it that you will attempt suicide someday?*). A recent validation study among Nigerian University students showed Cronbach's alpha of 0.80, indicating the SBQ‐R as a reliable screening tool in non‐clinical samples in West Africa (Aloba, Ojeleye, & Aloba, 2017). The SBQ‐R yields a total score between 3–18 points. For college students and undergraduates, a total score of 7 out of 18 on the SBQ‐R is the cut‐off (≥7) which indicates *high risk of suicidal behaviour, *whereas a total score between 3–6 indicates *low risk of suicidal behaviour* (Osman et al., 2001).

### 2.2 Data analysis

The answered questionnaires were checked for completeness, and data were entered into the Statistical Package for Social Sciences (SPSS version 21.0) for analysis. The list‐wise deletion of missing data approach was used, as the loss of cases due to missing data was <5% (Graham, 2009). Specifically, frequencies and cross‐tabulations were used to ascertain the prevalence estimates (and their overall 95% confidence intervals) of suicidal behaviours across the socio‐demographic characteristics of the participants. For further analysis, the socio‐demographic variables (shown in Table 1) were included as the correlates/explanatory variables, while the total score on the SBQ‐R, *suicidal behaviour risk*, was included as the outcome variable. The chi‐square (*χ* ^2^) test was used to examine possible relationships between the specified categorical explanatory variables and suicidal behaviour risk; Fisher's exact test was used where a cell expected frequency was less than five counts (Agresti, 2002; Kim, 2017). Point‐biserial correlation tests were performed to assess the associations between the continuous explanatory variables and suicidal behaviour risk (Prematunga, 2012). Given the general categorical nature of the data and the binary response format of the outcome variable (i.e., suicidal behaviour risk, scored *low* or *high*), binary logistic regression (Agresti, 2002) was used to ascertain possible associations between the correlates and the outcome variable. The candidate explanatory variables (i.e., socio‐demographic variables) were entered in the multivariable logistic regression model whether or not they had a statistically significant bivariate (*χ* ^2^) relationship with the outcome variable (Babyak, 2004; Peduzzi, Concato, Kemper, Holford, & Feinstein, 1996). Results of the logistic regression were reported as odds ratios with 95% confidence intervals. Statistically significant results were determined using the *p*‐value <0.05 (*p < *0.05).

### 2.3 Ethical statement

Prior to the administration of the questionnaire, all the students in each randomly selected class were informed about the study and their questions and concerns were addressed by the researchers. Each participant signed an actual consent form. Participation in this study was entirely voluntary. In keeping with the confidentiality and anonymous position of the study, the sitting arrangement was such that the students were made to sit far apart from each other to make it impossible for a participant to see how another participant responded to the survey. Tutors of the college present were not allowed into the classrooms where the administration of the survey was in session. Averagely, it took about 15 min to complete the questionnaire. This study received approval from the Centre for Suicide and Violence Research, Accra and permission from the Nurses and Midwifery College, Korle‐Bu, Accra, Ghana.

## 3 RESULT

The results of the data analysis are presented in relation to the prevalence estimates of suicidal behaviour and possible demographic correlates of suicidal behaviour risk.

### 3.1 Prevalence estimates of suicidal behaviours

Table 2 shows the univariate analysis (frequencies and proportions) of the demographic variables and suicidal behaviours: overall lifetime suicide ideation (15.4%; 95% CI = 0.11–0.20 [female = 16.2%; male = 7.1%]), lifetime suicide plans (6.6%; 95% CI = 0.04–0.10 [female = 6.9%; male = 3.6%]) and lifetime suicide attempt (2.3%; 95% CI = 0.01–0.05 [female = 2.2%; male = 3.6%]). Lifetime suicide threat was 13.4% (*N* = 41; female = 14.1%; male = 7.1%). It is worth noting that the participants in the suicide plan, suicide threat and suicide attempt subgroups might also have had suicide ideations.

**Table 2:** Lifetime prevalence estimates of suicidal behaviour (i.e., ideation, plan, threats and attempts), suicidal behaviour risk and chi‐square test

Variables	Ideation	Plan	Threat	Attempt	Suicidal behaviour risk		*χ* ^2^	*p *(2‐tailed)
					Low	High		
	47 (15.4%)	20 (6.6%)	41 (13.4%)	7 (2.3%)	279 (91.5%)	26 (8.5%)		
	*N* (%)	*N* (%)	*N* (%)	*N* (%)	*N* (%)	*N* (%)		
Gender								
Female	45 (16.2)	19 (6.9)	39 (14.0)	6 (2.2)	253 (91.3)	24 (8.7)	0.08	1.00
Male	2 (7.1)	1 (3.6)	2 (7.1)	1 (3.6)	26 (92.9)	2 (7.1)		
Religious groups								
Christian	45 (15.7)	19 (6.6)	39 (13.6)	7 (2.4)	263 (91.6)	24 (8.4)	0.28	0.65
Muslim	1 (5.9)	1 (5.9)	2 (11.8)	0	15 (88.2)	2 (11.8)		
Ethnic groups								
Akan	17 (12.3)	9 (6.5)	20 (14.5)	4 (2.9)	128 (92.8)	10 (7.2)	6.50	0.17
Ewe	14 (19.7)	5 (7.0)	9 (12.7)	0	68 (95.8)	3 (4.2)		
Ga‐Dangme	14 (21.2)	3 (4.5)	9 (13.6)	2 (3.0)	56 (84.8)	10 (15.2)		
Guan	0	1 (20.0)	1 (20.0)	1 (20.0)	4 (80.0)	1 (20.0)		
Other	1 (5.3)	1 (5.3)	1 (5.3)	0	17 (89.5)	2 (10.5)		
In romantic relationship								
No	23 (16.0)	12 (8.3)	16 (11.1)	5 (3.5)	132 (91.7)	12 (8.3)	0.03	1.00
Yes	24 (15.3)	7 (4.5)	24 (15.3)	2 (1.3)	143 (91.1)	14 (8.9)		
Sexual orientation								
Heterosexual	34 (14.1)	14 (5.8)	32 (13.3)	6 (2.5)	222 (92.1)	19 (7.9)	0.04	0.69
Non‐heterosexual	2 (9.1)	3 (13.6)	4 (18.2)	1 (4.5)	20 (90.9)	2 (9.1)		
Marital status								
Single	45 (15.6)	19 (6.6)	35 (12.2)	6 (2.1)	265 (92.0)	23 (8.0)	4.85	0.09
Married	0	0	3 (27.3)	1 (9.1)	10 (90.9)	1 (9.1)		
Other	2 (33.3)	1(16.7)	3 (50.0)	0	4 (66.7)	2 (33.3)		
Year of study								
1st year	16 (19.5)	7 (8.5)	13 (15.9)	1 (1.2)	76 (92.7)	6 (7.3)	0.52	0.77
2nd year	20 (16.5)	10 (8.3)	18 (14.9)	4 (3.3)	109 (90.1)	12 (9.9)		
3rd year	11 (10.8)	3 (2.9)	10 (9.8)	2 (2.0)	94 (92.2)	8 (7.8)		
Programme of study								
RGN	30 (18.8)	8 (5.0)	20 (12.5)	4 (2.5)	144 (90.0)	16 (10.0)	0.97	0.62
Midwifery	14 (16.9)	0	10 (12.0)	3 (3.6)	77 (92.8)	6 (7.2)		
RCHN	3 (4.8)	12 (19.4)	11 (17.7)	0	58 (93.5)	4 (6.5)		
School residential status								
Resident	44 (17.1)	10 (3.9)	32 (12.5)	7 (2.7)	235 (91.4)	22 (8.6)	0.003	1.00
Non‐resident	3 (6.3)	10 (20.8)	9 (18.8)	0	44 (91.7)	4 (8.3%)		
Truancy								
≤5 days	44 (15.3)	19 (6.6)	39 (13.5)	7 (2.4)	263 (91.3)	25 (8.7)	0.114	1.00
>5 days	3 (18.8)	1 (6.3)	2 (12.5)	0	15 (93.8)	1 (6.3)		

Note

RCHN: Registered Community Health Nursing; RGN: Registered General Nursing.

The 12‐month prevalence estimate of suicidal ideation was 21.3% (95% CI = 0.17–0.26 [female = 21.7%; male = 17.9%]). Furthermore, 2.0% of the participants (female = 2.2%; male = 0.0%) believed that it was likely that in the future they would attempt suicide. Again, 8.5% (95% CI = 0.06–0.12) of the nursing and midwifery college students surveyed met the criterion for the categorization as being at a *high risk *of suicidal behaviour (Table 2), indicating the need for further clinical assessment or referral to a mental health professional.

Relative to the Christian participants, the Muslims reported lower scores on suicidal ideations and plans (with no lifetime attempted suicide), but higher on suicidal behaviour risk (Muslim = 12.0%; Christian = 8.4%). Also, although more married students reported lifetime suicide attempts (9.1%) compared students who self‐identified as single (2.1%), in terms of suicidal behaviour risk, both groups were fairly similar, while students of *other* marital status scored the highest (33.3%) on suicidal behaviour risk. Students with non‐heterosexual orientation, married students and students of registered community health nursing (RCHN) all reported no likelihood of future suicidal behaviour. Students of RGN reported high suicidal behaviour risk (10.0%), compared with the midwifery (7.2%) and RCHN students (6.5%). The risk of suicidal behaviour was relatively similar across the three levels of study (1st year = 7.3%, 2nd year = 9.9% and 3rd year = 7.8%), although the 2nd‐year students were at a slightly higher risk. Similarly, students who identified as not in a romantic relationship were identified to be at a slightly higher risk of suicidal behaviour (5.0%) relative to those students who responded “yes” (3.2%) to being in a romantic relationship at the time of the study.

### 3.2 Socio‐demographic correlates of suicidal behaviour risk

The statistical test for possible univariate relationships (chi‐square test) between the socio‐demographic variables and suicidal behaviour risk found no statistically significant results (Table 2). A point‐biserial correlation was run to determine the relationship between age and suicidal behaviour risk; there was a statistically non‐significant positive correlation between age and suicidal behaviour risk (*r*_pb_ = 0.063, *N* = 305, *p* = 0.271).

In Table 2, several of the socio‐demographic variables showed sparse data bias (Greenland, Mansournia, & Altman, 2016) in terms of suicidal behaviour risk, mostly less than five cases in the *high* category. Thus, to avoid an unstable logistic regression model due to the sparse data problem, we applied the relaxed rule of at least five events per variable (Vittinghoff & McCulloch, 2007) to select the socio‐demographic variables included as correlates in the final logistic regression model. As shown in Table 3, the final logistic regression model was not statistically significant (*χ* ^2^_(_ *_df_*_ = 4)_ = 1.774, *p* = 0.777), accounting for 1.3% of the variance in the outcome variable (Nagelkerke *R* ^2^ = 0.013). In other words, the model did not show any statistically significant distinction between participants who scored low and high on suicidal behaviour risk. Thus, the prevalence of suicidal behaviour, differences and associations observed in this study is descriptive in nature rather than statistical.

**Table 3:** Logistic regression assessing the association between socio‐demographic variables and suicidal behaviour risk

Variables in model	*β*	*Wald*	*p‐value*	AOR	95% CI for AOR	
					Lower	Upper
Age	0.092	1.312	0.252	1.10	0.94	1.30
In romantic relationship	0.090	0.047	0.828	1.09	0.49	2.50
Year of study						
1st year	Reference					
2nd year	0.270	0.261	0.609	1.31	0.47	3.69
3rd year	0.009	0.000	0.987	1.01	0.33	3.07
Constant	−4.628	6.070	0.014	0.010		

Notes

AOR: adjusted odds ratio; CI: confidence interval; *β*: beta value.

Coding of variables in model: Age (coded continuously); In romantic relationship (No = 0, Yes = 1); Year of study (1 year = 0; 2nd year = 1; 3rd year = 2)

Model summary: *χ* ^2^_(_ *_df_*_ = 4)_ = 1.774, *p* = 0.777 (Cox & Snell *R* ^2^ = 0.006; Nagelkerke *R* ^2^ = 0.013; Homer & Lemeshow test = 0.918).

## 4 DISCUSSION

To our knowledge, this is the first study from Ghana that provides estimates of the prevalence of suicidal behaviour (ideations, plans, threats and attempts) and tries to document potential socio‐demographic correlates of the behaviour among nursing and midwifery college students.

### 4.1 Prevalence of suicidal behaviour

The main finding of this study is that 8.5% (female = 8.7%; male = 7.1%) of the 305 nursing and midwifery college students surveyed met the cut‐off criterion for the categorization as at high risk of suicidal behaviour. The overall lifetime prevalence estimates of suicide ideation (15.4%; female = 16.2%; male = 7.1%), plans (6.6%; female = 6.9%; male = 3.6%), threats (13.4%; female = 14.1%; male = 7.1%) and attempts (2.3%; female = 2.2%; male = 3.6%) and the 12‐month prevalence estimate of suicide ideation (21.3%; female = 21.7%; male = 17.9%) reported in this study are generally comparable to those found in high‐income countries, with more females than males reporting suicidal behaviour.. The 12‐month prevalence estimate of suicide ideation found in this study (21.3%) is comparable and quite consistent with the recent prevalence estimates among senior high school students in Ghana—18.2% (Asante et al., 2017; Baiden et al., 2019). More pointedly, the findings on the prevalence estimates of suicidal behaviour in this study are consistent with the global situation as found in the meta‐analysis by Mortier, Cuijpers et al. (2018). Put together, the prevalence estimates of suicidal behaviour and risk found in this study resonate with recent calls and campaigns from Ghana (Andoh‐Arthur, Asante, & Osafo, 2015) and elsewhere (Mortali & Moutier, 2018; Pace, Silk, Nazione, Fournier, & Collins‐Eaglin, 2018) for the improvement of on‐site counselling services to facilitate mental health help‐seeking by students on college campuses. To this end, emphasis has been placed on mental health literacy and stigma reduction, as doing this has the potential of encouraging students, particularly, males, to seek professional help (Rafal, Gatto, & DeBate, 2018).

It is evident in this study that more females than males reported both lifetime and 12‐month suicidal ideations, even though the suicidal behaviour risk between females and males is comparable (Table 2). This observation is consistent with previous studies in the area from high‐income countries (Aradilla‐Herrero et al., 2014; Leal & Santos, 2016) and among young people generally (Asante et al., 2017; Hawton et al., 2012; O'Connor et al., 2018), but contradicts the finding by Melissa‐Halikiopoulou et al. (2011) that lifetime suicidal ideations are higher among males than female nursing students in Greece.

Also, the students who reported as being in a romantic relationship scored lower on the suicidal behaviour risk, relative to those who reported as not in a romantic relationship. One potential explanation for this is that being in a relationship strengthens individuals’ sense of belongingness and could be a protective factor against suicidal behaviour. Additionally, meaningful social relationships represent an important avenue for support, which could help individuals deal with distress (Rhoades, Kamp Dush, Atkins, Stanley, & Markman, 2011). Generally, although the evidence presented in this study does not provide enough basis to attribute the reported prevalence estimates of suicidal behaviour to the family circumstances or even psychopathology of the participants, it may point to the general stressful nature of nursing and medical education in Ghana; admissions into government funded training institutions are fiercely competitive and once admitted a student must keep to the required performance to avoid being dismissed. Many students (who are often the first to go to college in their families) are expected by their families to be successful; students who are less resilient and lack strong support system may experience suicidal tendencies and other mental health challenges (Adu‐Gyamfi & Brenya, 2016; Evans‐Lacko & Thornicroft, 2019; Hakim et al., 2018).

### 4.2 Demographic correlates of suicidal behaviour

This study also attempted to describe some of the possible demographic correlates of suicidal behaviour among college nursing and midwifery students. However, the analysis showed no statistically significant associations, even though some odds ratios greater one (OR > 1) were obtained. The statistically insignificant results obtained in relation to the chi‐square tests and logistic regression analysis in this study may be attributable to three factors. The first plausible reason has to do with guarded responses or non‐disclosure mainly due to the general negative attitudes towards suicide and suicidal behaviour in Ghana. Attempted suicide is a crime in Ghana (Mishara & Weisstub, 2016); it is religiously sinful (Osafo et al., 2015), and morally, it is even tabooed to openly talk about suicide and suicidal behaviours (Sarpong, 2006). There is evidence to suggest that people found guilty of this code have been jailed or given hefty fines (Adinkrah, 2013). Knowledge of this law and social mores might have discouraged the participants from honestly reporting their histories of suicidal behaviours.

The second reason could be the possibility that as trainee healthcare professionals, the participants might be aware of the medical danger associated with attempting suicide, even though having suicidal ideations might seem less threatening. Previous studies have shown that among the pathways to suicidal death, the transition from suicidal ideations to death by suicide is less strong, compared with the transition from suicide attempts to death by suicide (Van Heeringen, Hawton, & Williams, 2000). Thus, our participants’ possible awareness of the strong connection between suicide attempt and death might account for the low estimates of suicide attempts in this study.

The third plausible reason is related to the sample size of this study. A cursory visual inspection of Table 2 shows frequent occurrence of zero cells and a relatively wide spread of sparse data. Widely spread sparse data lead to insignificant (or extremely significant) chi‐square results (Greenland et al., 2016; McHugh, 2013). Future studies may consider obtaining a relatively larger sample of participants to partly avoid the problem of sparse data.

### 4.3 Implications

This study has important implications for healthcare professionals and administration of nursing and midwifery training colleges (in Ghana) and research. An awareness of the suicidal behaviour risk and prevalence of suicidal behaviours among students should underscore the need for periodic screening of college students and the strengthening of on‐site student mental health and counselling services. Suicidal ideation should be a major concern for (mental) healthcare providers serving students and trainers of these students in colleges. In terms of research, the findings of this study could serve a useful beginning point for more detailed and broader research on suicidal behaviours among nursing and college students in Ghana.

### 4.4 Limitations

The findings of this research should not be taken without regard to the plausible limitations related to the study. First, the prevalence estimates may be an underestimation of the true extent of the problem among college students in Ghana. Previous studies have found non‐disclosure of suicidal behaviours among college students (De Luca, Yan, Lytle, & Brownson, 2014). This tendency of non‐disclosure, coupled with the highly stigmatized nature of suicidal behaviour in Ghana (Osafo et al., 2015), might have precluded our participants from fully reporting their history of suicidal behaviour, thereby providing socially desirable responses. Also, given that the data are cross‐sectional, causation of suicidal behaviours cannot be inferred. Additionally, the data for this study were collected from nursing and midwifery students from one training college, which limits the generalizability of the findings; hence, future studies can broaden the sample to include trainees from other nursing programmes and training colleges in the country. Despite these limitations, this study contributes pioneering descriptive evidence to our knowledge about the prevalence estimates of suicidal behaviour among nursing and midwifery college students in Ghana.

## 5 CONCLUSION

This study highlights the reality of suicidal behaviours among nursing and midwifery college students in Ghana and underscores the need to sensitize all stakeholders to promote mental health intervention efforts focusing on this group of students to improve early detection and treatment and support. The findings may also point to developing strategies and initiatives for suicide prevention on the campuses of nursing and midwifery training institutions in Ghana to building support networks and increasing awareness about the signs of suicidal behaviours.

## CONFLICT OF INTEREST

The authors declare that they have no conflict of interest.
